# Supplementary material for: A Pentavalent Shigella flexneri LPS-Based Vaccine Candidate Is Safe and Immunogenic in Animal Models
Source: Vaccines (Basel). 2023 Feb 3;11(2):345. doi: 10.3390/vaccines11020345 (PMC9966156; doi:10.3390/vaccines11020345)
Supplement: Supplementary file 1 [file vaccines-11-00345-s001.zip › vaccines-2131658-supplementary.pdf]

Table S1. Histopathological changes of internal organs after single or multiple subcutaneous injections with 125 μg dose of PLVF.

Organs and tissues	Histopathological changes	
	Acute toxicity test	Chronic toxicity test
Brain	NF	NF
Lymth nodes	NF	NF
Thyroid gland	NF	NF
Heart	NF	NF
Lung	NF	NF
Thymus	NF	NF
Stomach	NF	NF
Small intestine	NF	NF
Colon	NF	NF
Liver	NF	NF
Spleen	NF	NF
Kidney	NF	NF
Adrenal gland	NF	NF
Testicle	NF	NF
Ovary	NF	NF
Peyer's patches	NF	NF

Note: NF – not found

Brain

Lymth node

Thyroid gland

Heart

Lung

Thymus

Stomach

Small intestine

Colon

Liver

Spleen

Kidney

Adrenal gland

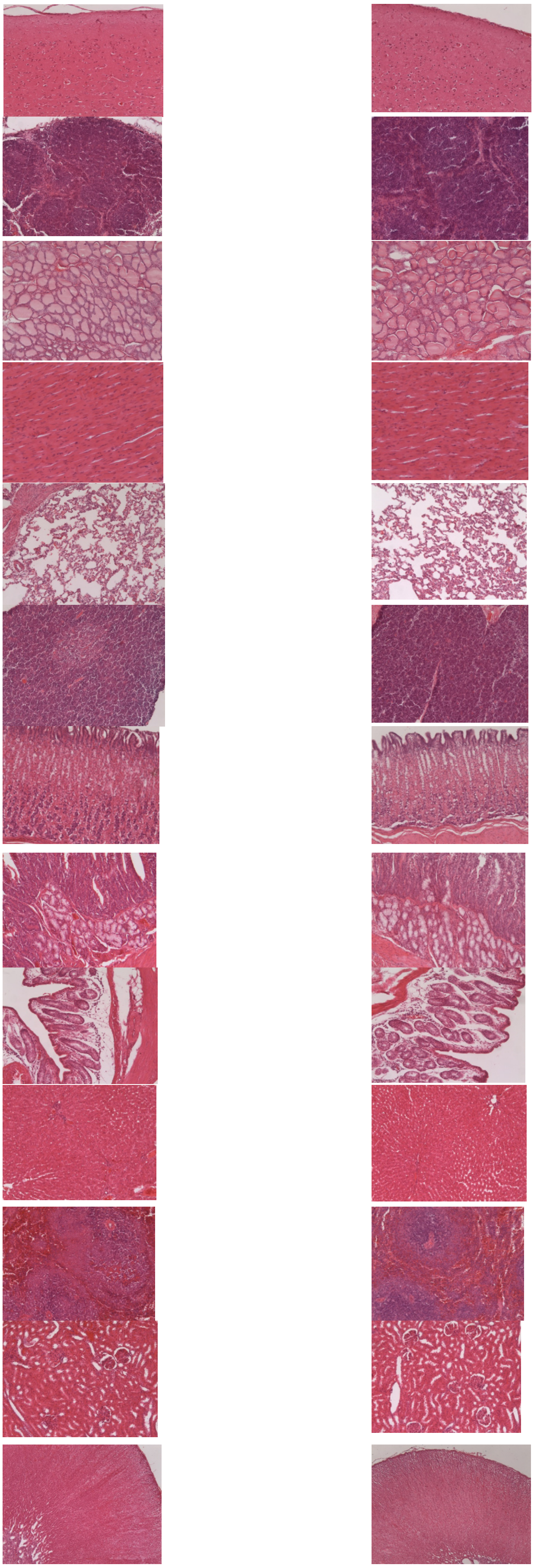

Testicle

Ovary

Peyer's patch

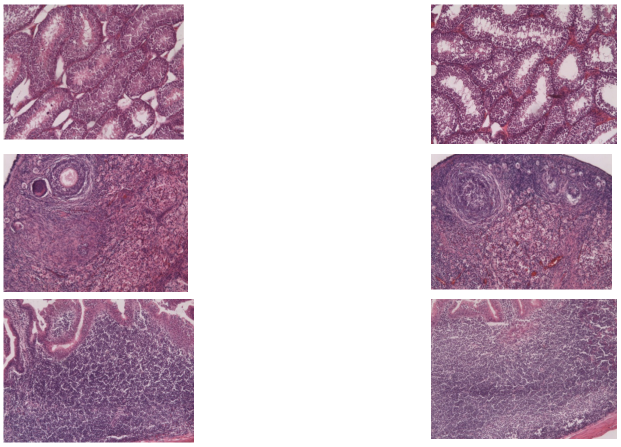

a b

Figure S1. Hematoxylin and eosin stained sections of the internal organs of rabbits from the control group (a) and 7 days after the last injection of PLVF (b). PLVF was administered subcutaneously at a dose of 125 µg daily for a week. Representative images from 5 groups

1b →2)-α-L-Rhap^{III}3,4Ac-(1→2)-α-L-Rhap^{II}-(1→3)-α-L-Rhap^{I}2Ac-(1→3)-β-D-Glc$p$NAc-(1→
α-D-Glc$p$-(1→4)┘

2a →2)-α-L-Rhap^{III}3,4Ac-(1→2)-α-L-Rhap^{II}-(1→3)-α-L-Rhap^{I}-(1→3)-β-D-Glc$p$NAc6Ac-(1→
α-D-Glc$p$-(1→4)┘

3a →2)-α-L-Rhap^{III}-(1→2)-α-L-Rhap^{II}-(1→3)-α-L-Rhap^{I}2Ac-(1→3)-β-D-Glc$p$NAc6Ac-(1→
└(3←1)-α-D-Glc$p$

Y →2)-α-L-Rhap^{III}3,4Ac-(1→2)-α-L-Rhap^{II}-(1→3)-α-L-Rhap^{I}-(1→3)-β-D-Glc$p$NAc6Ac-(1→

6 →2)-α-L-Rhap^{III}3,4Ac-(1→2)-α-L-Rhap^{II}-(1→4)-β-D-Glc$p$A-(1→3)-β-D-Gal$p$NAc-(1→

Figure S2. Chemical structure of the repeating unit of the O-specific polysaccharide chain of *S. flexneri* serotypes 1b, 2a, 3a, 6, and Y.
